# Supplementary material for: Illumination of a progressive allosteric mechanism mediating the glycine receptor activation
Source: Nat Commun. 2023 Feb 13;14:795. doi: 10.1038/s41467-023-36471-7 (PMC9925812; doi:10.1038/s41467-023-36471-7)
Supplement: Supplementary file 1 — Supplementary Information [file 41467_2023_36471_MOESM1_ESM.pdf]

Supplementary Figures

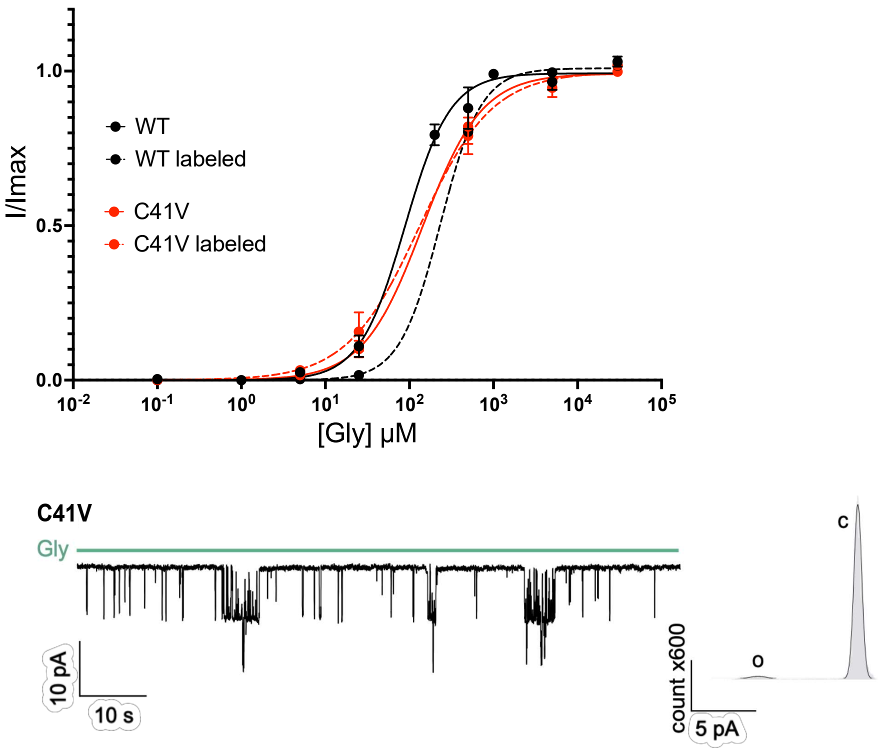

Supplementary Figure 1. Control experiments showing labeled and unlabeled WT and C41V background mutant.

Upper panel: current dose-response curves with mean ± the S.E.M. of the WT (black) and C41V (red) mutant without labeling in solid line and with MTS-TAMRA labeling in dotted line (n=4). The labeling of the WT triggers a weak loss of function of the receptor, while the C41V mutant is not impacted by the labeling. Lower left panel: single channel recording obtained by outside-out patch-clamp on HEK293 cells of the C41V mutant evoked by glycine application. The C41V mutant displays wild-type like glycine-elicited dose-response curve (TEVC) and unitary conductance as studied by outside-out single channel recording (Bormann *et al.*, 1987 and 1993). Lower right panel: histograms of current amplitude representing the closed state (c) and the open state (o).

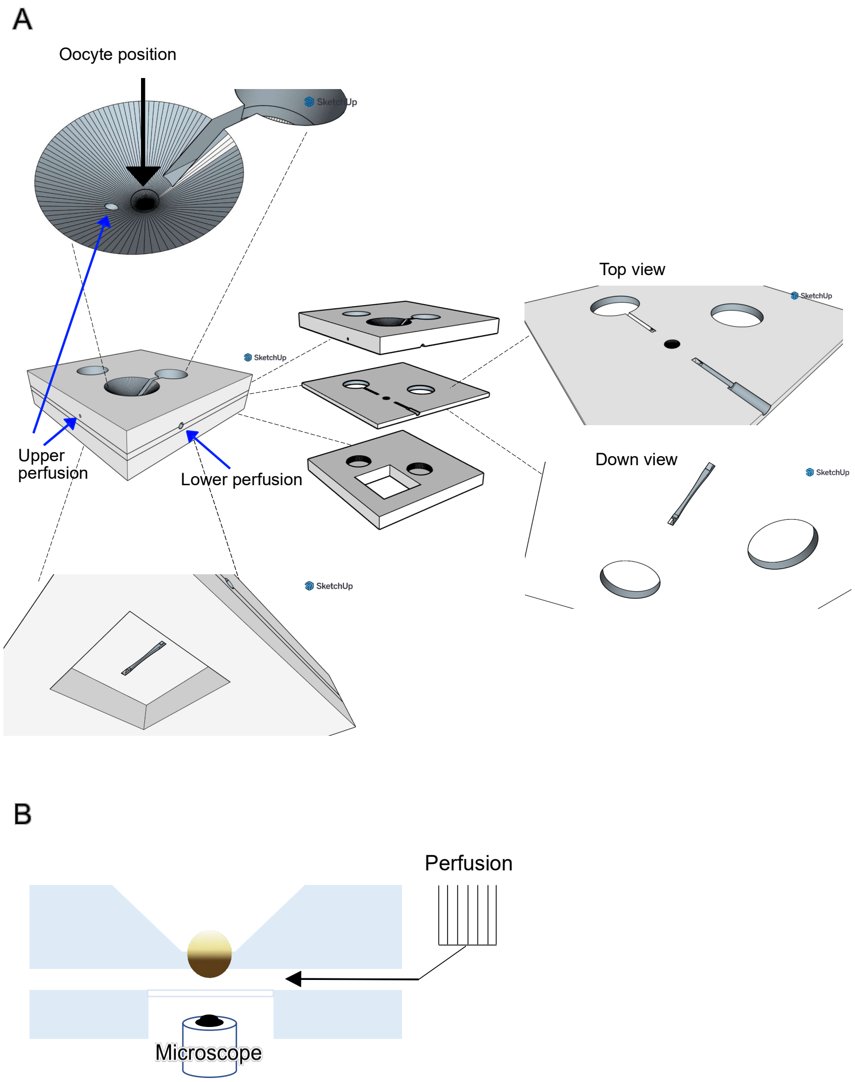

Supplementary Figure 2. Blueprint of the perfusion recording chamber and schematic view of the voltage-clamp fluorometry set-up used in this study.

(**A)** The recording chamber has been designed to perfuse only the portion of the animal pole that is imaged by fluorescence. A venturi effect allows to seal the oocyte in the chamber without any activation of receptors expressed on the upper side of the oocyte. **(B)** Schematic view of the voltage-clamp fluorometry set-up used in this study.

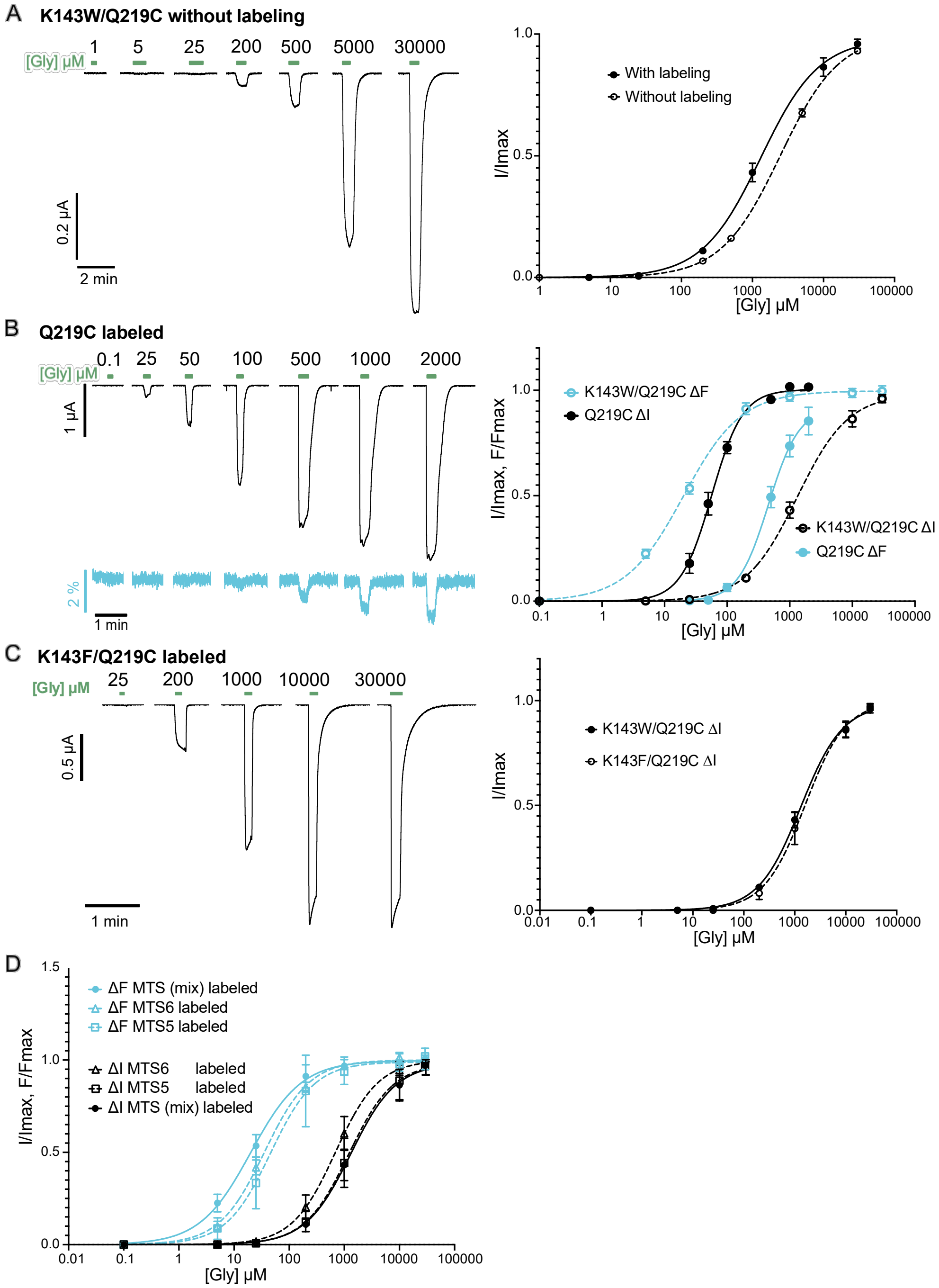

Supplementary Figure 3. Control experiments showing labeled Q219C, unlabeled K143W/Q219C, labeled K143F/Q219C and MTS-5/6-TAMRA VCF data on C41V GlyR.

(A) Left panel: representative current recording of K143W/Q219C without labeling. Right panel: dose-response curves with mean ± the S.E.M. of K143W/Q219C labeled with MTS-TAMRA (solid line) and unlabeled (dotted line, n=5). The labeling of the receptors triggers a small gain-of-function. **(B)** Left panel: representative VCF recordings of Q219C labeled with MTS-TAMRA. Fluorescence variation reaches 1.97 ± 0.49 % of ΔF/Fmax (n=6). Right panel: VCF glycine dose-response curves with mean ± the S.E.M. of Q219C labeled with MTS-TAMRA (solid line, n=6) compared to the K143W/Q219C (dotted line). For Q219C, the fluorescence (cyan) is shifted to a higher concentration of glycine compared to the current variation (black), with an EC_{50}^{fluo} that is 10-fold higher than the $EC_{50}^{current}$. (**C**) left panel: representative current recording of K143F/Q219C labeled with MTS-TAMRA. Right panel: VCF glycine dose-response curves with mean ± the S.E.M. of K143W/Q219C labeled with MTS-TAMRA (solid line, n=5) compared to the K143F/Q219C (dotted line, n=5). No fluorescence variation is elicited by gycine for the mutant K143F/Q219C. **(D)** Fluorescence (cyan) and current (black) dose-response curves with mean ± the S.E.M. of mutant K143W/Q219C labeled with isomers of MTS-TAMRA (solid line, round point), MTS-6-TAMRA (dotted line, triangular point, n=5) and MTS-5-TAMRA (dotted line, rectangular point, n=5).

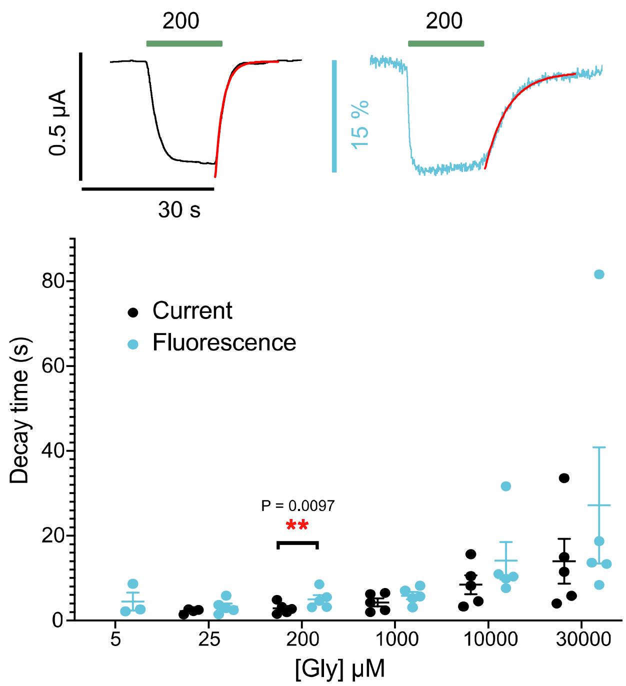

Supplementary Figure 4. Single exponential fitting of the current and fluorescence traces offset of K143W/Q219C sensor on C41V GlyR.

Upper panel: single exponential fitting (red line) of the current and fluorescence traces offset. Lower panel: time constants τ (offset) values obtained via single exponential fitting with mean and S.E.M (n=3 and 4 for glycine concentrations under 25 µM and n=5 for other concentrations). error bars at different glycine concentrations. Unpaired two-sided student t-test indicates the significance of the difference between fluorescence and current offset (**: *P* < 0,005; ***: *P* < 0,0005).

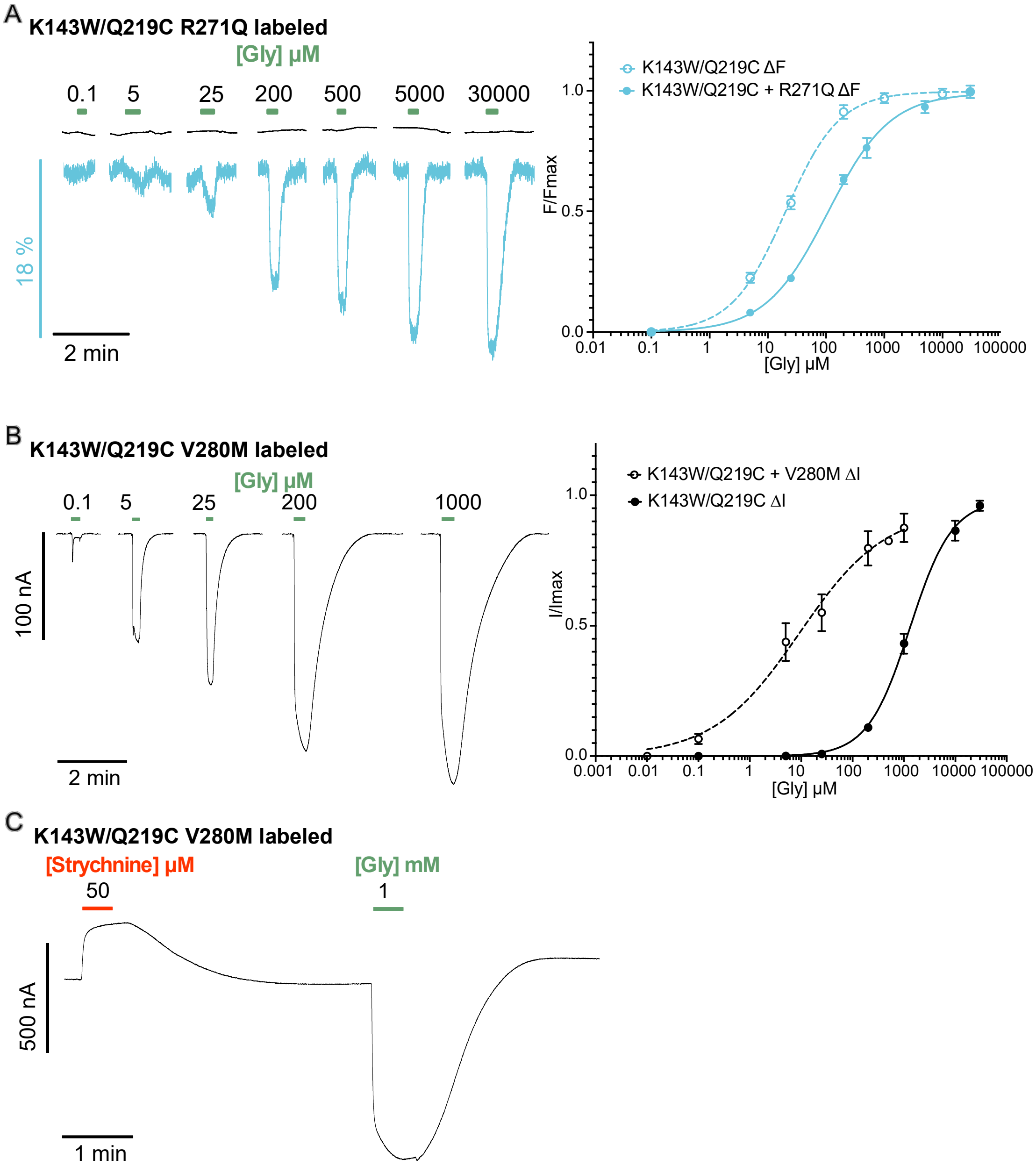

Supplementary Figure 5. VCF experiments of hyperekplexia mutations on C41V GlyR.

(A) Left panel: representative VCF recordings of K143W/Q219C/R271Q labeled with MTS-TAMRA (no currents observed in this construct). Fluorescence variation reaches 1.97 ± 0.49 % of ΔF/Fmax (n=6). Right panel: VCF glycine dose-response curves with mean ± the S.E.M. of K143W/Q219C/R271Q labeled with MTS-TAMRA (solid line, n=6) compared to the K143W/Q219C (dotted line). **(B)** Left panel: representative VCF recording of K143W/Q219C/V280M labeled with MTS-TAMRA. Note that no change in fluorescence is observed in this construct. Right panel: dose-response curves with mean ± the S.E.M. of K143W/Q219C (solid line) and K143W/Q219C/V280M labeled with MTS-TAMRA (dotted line, n=5). (**C**) Representative VCF recording of K143W/Q219C/V280M under strychnine (red) application compared to glycine(green) application. The currents inhibited by strychnine produce a 31.14 ± 6.72% inhibition current compared to the maximum current elicited by 1mM glycine.

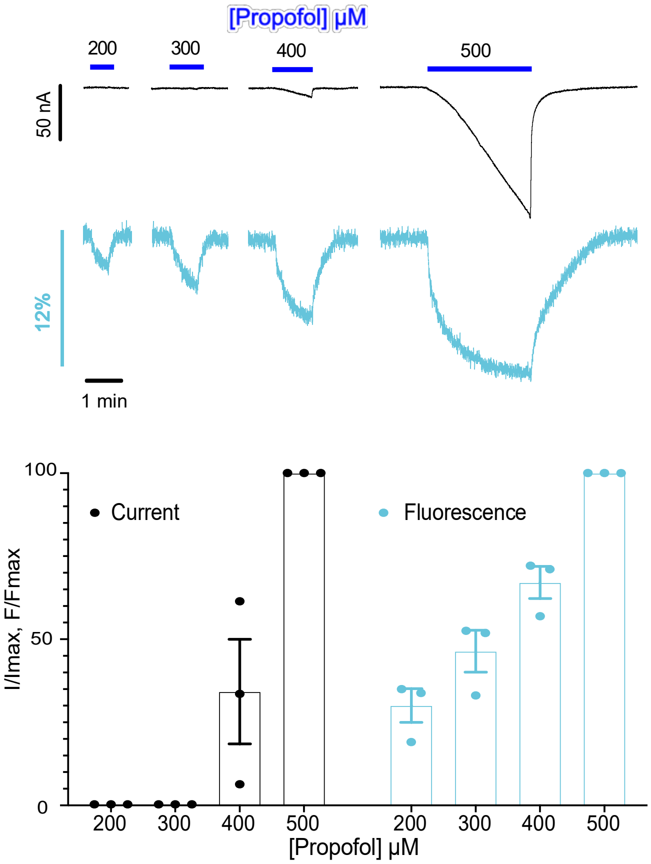

Supplementary Figure 6. Electrophysiological and fluorescence characterization of K143W/Q219C sensor on C41V GlyR with propofol.

Upper panel: representative VCF recording under different concentration (200 to 500 µM) of propofol application. Lower panel: fluorescence (cyan) and current (black) variations normalized to the fluorescence and current variations under 500 µM of propofol (mean and S.E.M. (n=3). At low concentrations of propofol (less than 300 µM), a fluorescence variation is observed without any current. Of note, the slow kinetics of the fluorescence and current traces is likely due to the slow kinetics of partition of propofol into the plasma membrane (Heusser *et al*., 2018).

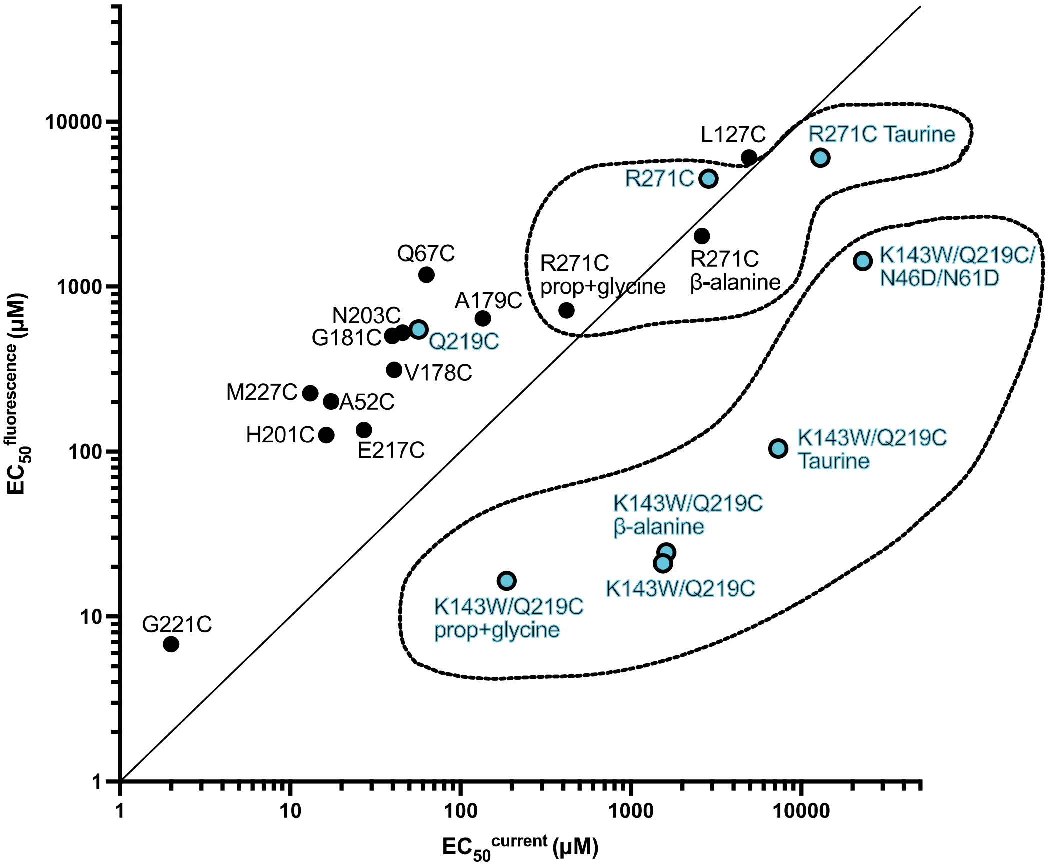

Supplementary Figure 7. plot representing the $EC_{50}^{fluorescence}$ as a function of the $EC_{50}^{current}$ of labeled cysteine mutants from VCF data

Labeled mutants that are colored black are taken from previously published articles (Pless et al J. Biol. Chem 282, 36057–36067 (2007), JBC 284, 15847–15856 (2009), JBC 284, 27370–27376 (2009), and those colored cyan are from this work. The data points correspond to activation by glycine, unless when other ligands are specified. Mutants showing $EC_{50}^{fluorescence}/EC_{50}^{current}$ values lower than 1, which are indicative of an intermediate phenotype, are located below the diagonal. The plot shows that the vast majority of the Pless mutants do not display an intermediate phenotype, with the exception of R271C activated by the partial agonists beta-alanine and taurine. The plot shows that most mutants displaying a $EC_{50}^{fluorescence}/EC_{50}^{current}$ higher than 1 display an $EC_{50}^{current}$ in the WT-range, while those displaying a $EC_{50}^{fluorescence}/EC_{50}^{current}$ lower than 1 (indicative of an intermediate phenotype), are characterized by a marked loss of function, with the exception of the condition in glycine plus propofol (on K143W/Q219C).

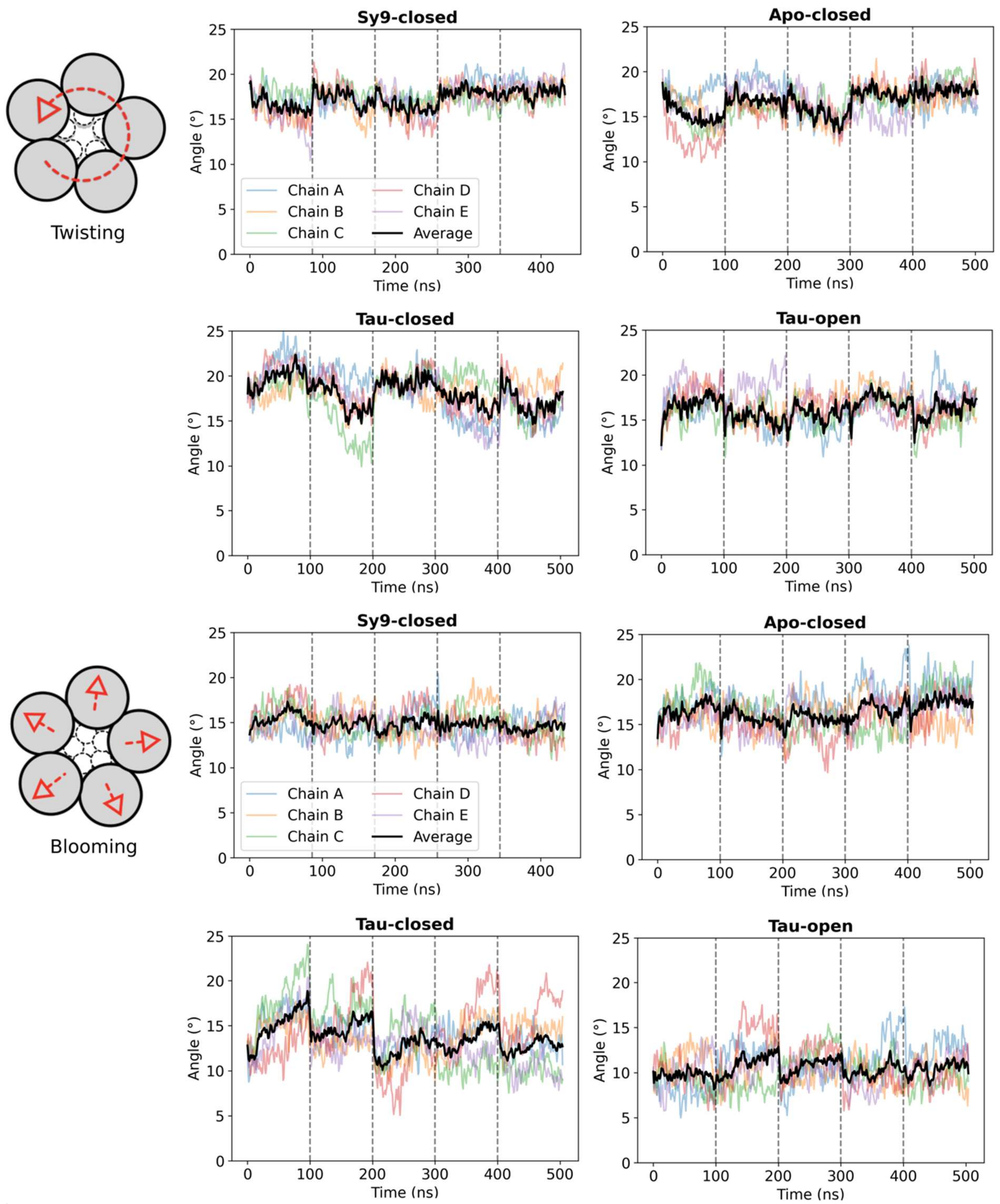

Supplementary Figure 8. Twisting and blooming angles in MD simulations of the wild type (WT). The time series of the twisting angle (upper panel) and the blooming angle (lower panel) are shown for each set of simulations (i.e., apo-closed, sy9-closed, tau-closed and tau-open). Each set includes five independent replicas whose time series are separated by dashed vertical lines. Color-coded lines correspond to individual subunits, the thick black line shows the average over the pentamer. Concerning receptor twisting (upper panel), no clear trend is observed. The average twisting angle fluctuates from 16° to 23° in the closed-channel conformations and is slightly lower in tau-open. The blooming angle (lower panel) is more informative. Its average value is stable around 10° in tau-open, while it increases to 16° in sy9-closed and apo-closed. In tau-closed, receptor blooming is more pronounced in replicas 1 and 2 due to the spontaneous unbinding of taurine (two unbinding events in replica 1, and one in replica 2), whereas it remains close to 13° in the other replicas. Overall, this analysis suggests that receptor's un-blooming, i.e., the compaction of the ECD, is associated to agonist binding, as seen in the apo-closed versus tau-closed and tau-open simulations. Additionally, an increase of the blooming angle in tau-closed is observed because of taurine unbinding. Finally, the active state represented by tau-open features a stable un-bloomed conformation.

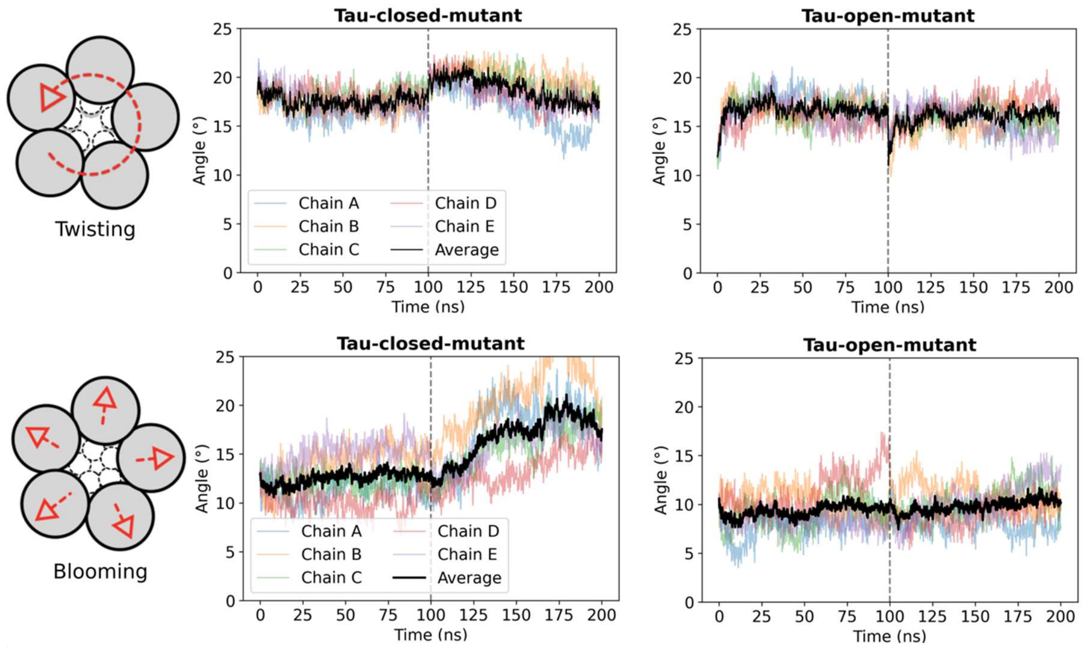

Supplementary Figure 9. Twisting and blooming angles in MD simulations of the triple mutant (K143W/Q219C/C41V). The time series of twisting angle (upper panel) and the blooming angle (lower panel) are show for each set of simulations (tau-closed and tau-open). Each set includes two independent replicas whose time series are separated by dashed vertical lines. Color-coded lines correspond to individual subunits, the thick black line shows the average over the pentamer. The behaviour of the triple-mutant simulations is WT-like. The twisting angle does not show any trend, being slightly higher in the tau-close simulations (around 17.5°) than in tau-open (around 16.3°). Regarding the blooming angle, the difference between two sets of simulations is more pronounced. The average for tau-closed is 15.2° while for tau-open it fluctuates around 9.7°. As for WT, spontaneous taurine unbinding was observed in replica 2 (Fig.S9). The impact of taurine unbinding is shown by the blooming angle, which increases through time in replica 2, while it stays stable in replica 1. Overall, the simulation results for the mutant are like those collected for WT. Therefore, the WT simulations presented in this manuscript are appropriate to explore the conformational dynamics of the mutant used in the VCF experiments.

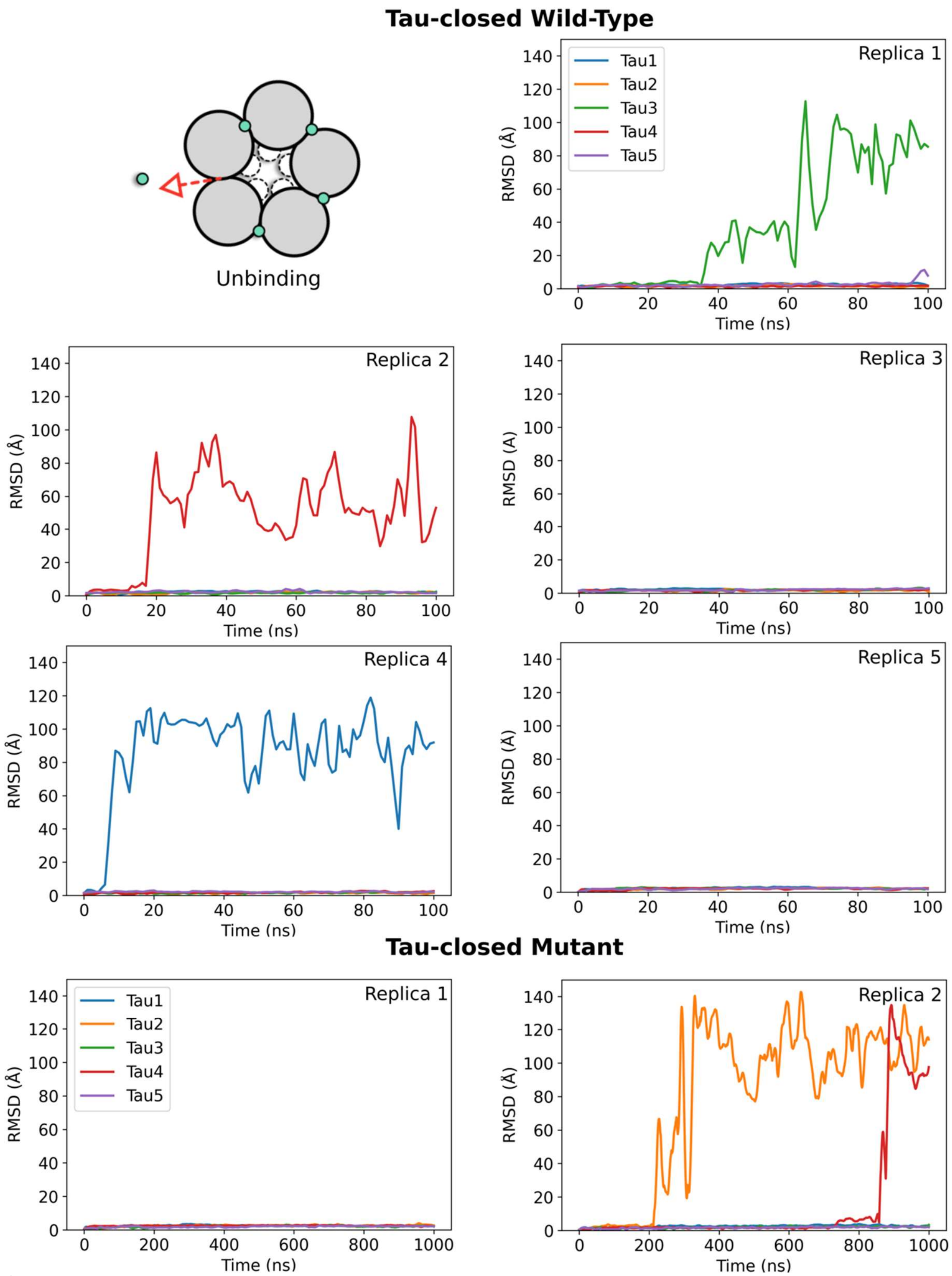

Supplementary Figure 10. Spontaneous unbinding of taurine in MD simulations of the tau-closed state. The RMSD of taurine was monitored over time without optimal superimposition of the atomic coordinates to account for both translational and rotational motions of the ligand. If the RMSD is small and stable, taurine remains bound. When the RMSD increases suddenly, taurine unbinds. Results for the WT simulations are shown on top, those for the K143W/Q219C/C41V triple mutant on bottom. In the WT simulations, two taurine ligands unbind in replica 1, one in replica 2, and one in replica 4. In the mutant simulations, two taurine unbind in replica 2 and none in replica 1.

Supplementary Table 1. EC_{50} values for current and fluorescence responses to β-alanine and taurine at labeled K143W/Q219C and R271C mutants on C41V GlyR.

K143W/Q219C	$EC_{50}^{current}$ (µM)	n_H	EC_{50}^{fluo} (µM)	n_H	n
Taurine	7325.35 ± 2442.41	0.78 ± 0.11	104.46 ± 18.28	0.88 ± 0.05	6
β-alanine	1615.78 ± 502.42	1.22 ± 0.39	24.51 ± 5.96	0.83 ± 0.10	6
Propofol + glycine	186.59 ± 40.49	1.38 ± 0.13	16.49 ± 6.82	1.73 ± 0.78	6

R271C	$EC_{50}^{current}$ (µM)	n_H	EC_{50}^{fluo} (µM)	n_H	n
Taurine	12972.00 ± 2364.96	1.24 ± 0.24	6044.00 ± 1054.84	1.36 ± 0.32	5

Supplementary Table 2. ΔFmax/F (%) and ΔFmax/ΔImax (%) values to glycine for C41V GlyRα1 mutants.

K143W/Q219C	ΔFmax/F (%)	ΔFmax/ΔImax (%)
Q219C	1.97 ± 0.21	0.21 ± 0.03
Q219C/K143W	12.2 ± 1.03	1.27 ± 0.43
Q219C/K143W/N46D/N61D	15.19 ± 5.14	4.15 ± 0.76

Supplementary Table 3. τ_{rise} and τ_{decay} values for fluorescence and current at labeled K143W/Q219C on C41V GlyR.

Risetime

concentration (µM)	τ current (s)	τ fluorescence (s)
5		7.10 ± 2.64
25	4.95 ± 0.43	2.43 ± 0.29
200	4.51 ± 1.18	1.22 ± 0.27
1000	2.46 ± 0.34	0.68 ± 0.10
10000	1.24 ± 0.12	0.43 ± 0.04
30000	1.29 ± 0.26	0.51 ± 0.08

Decaytime

concentration (µM)	τ current (s)	τ fluorescence (s)
5		4,00 ± 1,56
25	2.20 ± 0.28	3.28 ± 0.83
200	2.88 ± 0.59	5.00 ± 1.11
1000	4.28 ± 0.94	5.86 ± 0.96
10000	8.45 ± 2.21	14.11 ± 4.95
30000	13.98 ± 5.28	27.15 ±15.34

Supplementary Table 4. Cα-RMSD, blooming angle and twisting angle average values for the WT and triple-mutant MD simulations. Only the last 80 ns of simulation were considered.

System	RMSD (Å)	Blooming angle (°)	Twist angle (°)
Tau_open WT	1.62 +/- 0.02	10.61 +/- 0.30	16.51 +/- 0.34
Tau_closed WT	1.53 +/- 0.02	14.04 +/- 0.58	17.83 +/- 0.88
Apo_closed WT	1.58 +/- 0.03	16.52 +/- 0.38	16.60 +/- 0.50
Sy9_closed WT	1.35 +/- 0.06	15.02 +/- 0.20	17.41 +/- 0.34
Tau_open Mutant	1.63 +/- 0.07	9.70 +/- 0.24	16.30 +/- 0.57
Tau_closed Mutant	1.64 +/- 0.14	15.20 +/- 0.47	17.54 +/- 0.31
