# Supplementary material for: Illumination of a progressive allosteric mechanism mediating the glycine receptor activation
Source: Nat Commun. 2023 Feb 13;14:795. doi: 10.1038/s41467-023-36471-7 (PMC9925812; doi:10.1038/s41467-023-36471-7)
Supplement: Supplementary file 3 — Reporting Summary [file 41467_2023_36471_MOESM3_ESM.pdf]

nature portfolio

Corresponding author(s): Pierre-Jean Corringer
Marco Cecchini

Last updated by author(s): 09/01/2023

Reporting Summary

Nature Portfolio wishes to improve the reproducibility of the work that we publish. This form provides structure for consistency and transparency in reporting. For further information on Nature Portfolio policies, see our Editorial Policies and the Editorial Policy Checklist.

Statistics

For all statistical analyses, confirm that the following items are present in the figure legend, table legend, main text, or Methods section.

n/a	Confirmed	
☐	☒	The exact sample size (n) for each experimental group/condition, given as a discrete number and unit of measurement
☐	☒	A statement on whether measurements were taken from distinct samples or whether the same sample was measured repeatedly
☐	☒	The statistical test(s) used AND whether they are one- or two-sided<br>*Only common tests should be described solely by name; describe more complex techniques in the Methods section.*
☒	☐	A description of all covariates tested
☒	☐	A description of any assumptions or corrections, such as tests of normality and adjustment for multiple comparisons
☐	☒	A full description of the statistical parameters including central tendency (e.g. means) or other basic estimates (e.g. regression coefficient) AND variation (e.g. standard deviation) or associated estimates of uncertainty (e.g. confidence intervals)
☒	☐	For null hypothesis testing, the test statistic (e.g. F, t, r) with confidence intervals, effect sizes, degrees of freedom and P value noted<br>*Give P values as exact values whenever suitable.*
☒	☐	For Bayesian analysis, information on the choice of priors and Markov chain Monte Carlo settings
☒	☐	For hierarchical and complex designs, identification of the appropriate level for tests and full reporting of outcomes
☒	☐	Estimates of effect sizes (e.g. Cohen's d, Pearson's r), indicating how they were calculated

Our web collection on statistics for biologists contains articles on many of the points above.

Software and code

Policy information about availability of computer code

Data collection	Software for two-electrode voltage-clamp and voltage-clamp fluorometry data acquisition: Clampex (Axon pClamp 10.6) from Molecular devices.<br>Software for outside-out data acquisition: Clampex (Axon pClamp 10.5) from Molecular devices.<br>Software for molecular dynamics simulations: CHARMM-GUI, GROMACS 2021.4.<br>Software for docking: QuickVina 2.1 and MGLTools.<br>Software for structure visualization: PyMol.
Data analysis	Software for electrophysiological data analysis: Clampfit (Axon pClamp 10.6) from Molecular devices.<br>Software for statistical analysis: GraphPad Prism 9.3.1 and Python.<br>Software for Blooming and twisting angles analysis: WOROM (https://sourceforge.net/p/wordom/codehg/ci/default/tree/).<br>Software for MD analysis: MDAnalysis.

For manuscripts utilizing custom algorithms or software that are central to the research but not yet described in published literature, software must be made available to editors and reviewers. We strongly encourage code deposition in a community repository (e.g. GitHub). See the Nature Portfolio guidelines for submitting code & software for further information.

Data

Policy information about availability of data

All manuscripts must include a data availability statement. This statement should provide the following information, where applicable:

- Accession codes, unique identifiers, or web links for publicly available datasets
- A description of any restrictions on data availability
- For clinical datasets or third party data, please ensure that the statement adheres to our policy

The data that support the findings of this study are available from the corresponding author upon reasonable request and from the data source provided for the article.

Human research participants

Policy information about studies involving human research participants and Sex and Gender in Research.

Reporting on sex and gender	*Use the terms sex (biological attribute) and gender (shaped by social and cultural circumstances) carefully in order to avoid confusing both terms. Indicate if findings apply to only one sex or gender; describe whether sex and gender were considered in study design whether sex and/or gender was determined based on self-reporting or assigned and methods used. Provide in the source data disaggregated sex and gender data where this information has been collected, and consent has been obtained for sharing of individual-level data; provide overall numbers in this Reporting Summary. Please state if this information has not been collected. Report sex- and gender-based analyses where performed, justify reasons for lack of sex- and gender-based analysis.*
Population characteristics	*Describe the covariate-relevant population characteristics of the human research participants (e.g. age, genotypic information, past and current diagnosis and treatment categories). If you filled out the behavioural & social sciences study design questions and have nothing to add here, write "See above."*
Recruitment	*Describe how participants were recruited. Outline any potential self-selection bias or other biases that may be present and how these are likely to impact results.*
Ethics oversight	*Identify the organization(s) that approved the study protocol.*

Note that full information on the approval of the study protocol must also be provided in the manuscript.

Field-specific reporting

Please select the one below that is the best fit for your research. If you are not sure, read the appropriate sections before making your selection.

☒ Life sciences ☐ Behavioural & social sciences ☐ Ecological, evolutionary & environmental sciences

For a reference copy of the document with all sections, see nature.com/documents/nr-reporting-summary-flat.pdf

Life sciences study design

All studies must disclose on these points even when the disclosure is negative.

Sample size	All voltage-clamp experiments were performed with Xenopus laevis oocytes from at least two different batches (oocytes obtained from ovaries of at least two different animals). Oocytes from different batches were recorded in independent set of experiments. For each batch, oocytes expressing wild-type Glycine receptor were recorded as a control for mutants tested.
Data exclusions	Criteria for data exclusion were the following: 1) When the leak current is higher than 2 µA. 2) When the peak current size is insufficient for robust analysis. 3) When the baseline of the recording in unstable. 4) Due to the specificity of the recording chamber, some data representing a current with a rebound during the perfusion are also excluded (showing that the oocyte moved during the perfusion). 5) When the fluorescence signal is not enough for robust analysis. Approximately 20% of correct electrophysiological recordings were excluded.
Replication	We ensure to have at least one cell for a recorded batch and ultimately obtain at least 5 cells from 2 or 3 different batches. As internal control, we record some oocytes expressing the wild-type Glycine receptor perfused with 3 different concentrations of glycine to check the current EC50.
Randomization	Randomization is not relevant. (the experimental conditions were the same for each oocyte).
Blinding	Not relevant in our study: no conclusion is drawn from potential differences between constructs displaying similar phenotypes.

Reporting for specific materials, systems and methods

We require information from authors about some types of materials, experimental systems and methods used in many studies. Here, indicate whether each material, system or method listed is relevant to your study. If you are not sure if a list item applies to your research, read the appropriate section before selecting a response.

Materials & experimental systems

n/a	Involved in the study
☒	☐ Antibodies
☒	☐ Eukaryotic cell lines
☒	☐ Palaeontology and archaeology
☐	☒ Animals and other organisms
☒	☐ Clinical data
☒	☐ Dual use research of concern

Methods

n/a	Involved in the study
☒	☐ ChIP-seq
☒	☐ Flow cytometry
☒	☐ MRI-based neuroimaging

Animals and other research organisms

Policy information about studies involving animals; ARRIVE guidelines recommended for reporting animal research, and Sex and Gender in Research

Laboratory animals	We used oocytes form Xenopus laevis. Ovaries were obtained from Portsmouth European Xenopus resource center (Portsmouth, United Kingdom) and Ecocyte Biosciences (Dortmunf, Germany). No animals were handled by the authors.
Wild animals	No wild animals were used.
Reporting on sex	No report about the sex: oocytes are unfertilized.
Field-collected samples	No field-collected samples were used.
Ethics oversight	Oocytes were provided by two European Xenopus laevis center Portsmouth European Xenopus resource center and Ecocyte Biosciences.

Note that full information on the approval of the study protocol must also be provided in the manuscript.
